# A Hybrid Machine Learning Approach for Structure Stability Prediction in Molecular Co-crystal Screenings

**DOI:** 10.1021/acs.jctc.2c00343

**Published:** 2022-06-16

**Authors:** Simon Wengert, Gábor Csányi, Karsten Reuter, Johannes T. Margraf

**Affiliations:** † Fritz-Haber-Institut der Max-Planck-Gesellschaft, Faradayweg 4-6, 14195 Berlin, Germany; ‡ Chair of Theoretical Chemistry, Technische Universitát München, 85747 Garching, Germany; § Engineering Laboratory, University of Cambridge, Cambridge CB2 1PZ, United Kingdom

## Abstract

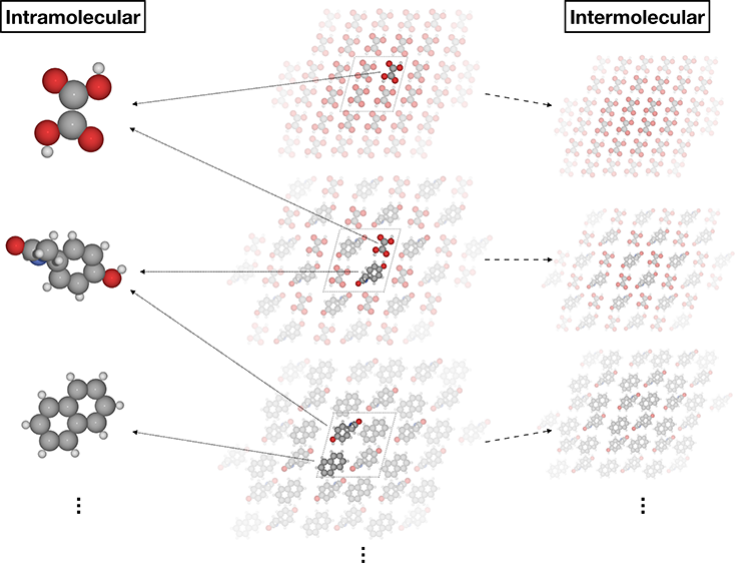

Co-crystals are a highly interesting material class as varying their components and stoichiometry in principle allows tuning supramolecular assemblies toward desired physical properties. The *in silico* prediction of co-crystal structures represents a daunting task, however, as they span a vast search space and usually feature large unit cells. This requires theoretical models that are accurate and fast to evaluate, a combination that can in principle be accomplished by modern machine-learned (ML) potentials trained on first-principles data. Crucially, these ML potentials need to account for the description of long-range interactions, which are essential for the stability and structure of molecular crystals. In this contribution, we present a strategy for developing Δ-ML potentials for co-crystals, which use a physical baseline model to describe long-range interactions. The applicability of this approach is demonstrated for co-crystals of variable composition consisting of an active pharmaceutical ingredient and various co-formers. We find that the Δ-ML approach offers a strong and consistent improvement over the density functional tight binding baseline. Importantly, this even holds true when extrapolating beyond the scope of the training set, for instance in molecular dynamics simulations under ambient conditions.

## 1 Introduction

The physical properties of a molecular crystal are strongly dependent on the arrangement of its building blocks in the solid state.^1^ In aggregate-induced emission, for instance, interactions in the crystalline phase (or even in concentrated solution) cause otherwise non-luminescent molecules to become emissive.^2^ Similarly, piezochromic luminescent materials change the color of their emission when intermolecular arrangements in the solid state are altered by external mechanical stimuli.^3^ Beyond these specific examples, the large variety of crystal forms detected and characterized for certain molecules reveals that the crystal structure impacts many other properties as well, such as aqueous solubility,^4^ charge transport,^5^ or plastic deformation^6^ to name but a few.

Being able to control molecular arrangements in the solid state, consequently, enables tuning materials toward desired properties.^7^ The design of multi-component molecular crystals, so-called co-crystals, is promising in this respect as it provides a versatile route to this goal.^8^ Here, the molecule of interest crystallizes in the presence of another compound, a so-called co-former. Co-crystallization has garnered interest in both academia and industry as a strategy for the design of materials with improved performance. Applications include non-linear optics,^9^ energetic materials,^10^ and, most notably, pharmaceuticals.^11^ Here, active pharmaceutical ingredients are often combined with co-formers to improve their bioavailabilty (e.g., by tuning the dissolution rate, solubility, compressibility, and thermal stability of the co-crystal).^12,13^

The space of possible co-formers is generally quite large. For pharmaceuticals, the “generally regarded as safe” (GRAS) list is often used, which contains hundreds of molecules considered as safe for human consumption. The synthesis of multi-component crystals thus provides a large design space. Unfortunately, the successful formation of a co-crystal from its compounds is by no means trivial.^14^ Indeed, recrystallization is actually a common technique for purifying compounds, i.e., to separate them from one another. Moreover, the stability and structure of a potential co-crystal are hard to predict as they result from a delicate balance between relatively weak interactions.^15^ Unlike conventional covalent chemistry, the synthesis of co-crystals is thus much more difficult to plan and often a game of trial and error. A more targeted approach would therefore be highly desirable. Here, computational methods could play an important role, e.g., by predicting whether a given co-former will lead to stable co-crystals and which structural motifs are likely to be formed for a given combination. This would allow narrowing the list of potential co-formers down to a few promising candidates and thus dramatically reduce the number of necessary experiments and associated costs.

The *in silico* search for molecular crystal structures faces some major challenges, however.^16^ On one hand, the large search space of potential structures requires evaluating the stability of a large number of trial crystals. On the other hand, highly accurate (and thus computationally expensive) levels of theory need to be applied for a reliable prediction of crystal lattice energies.^17^ Even for single-component crystals, this leads to a difficult trade-off between adequately exploring the space of possible structures and using sufficiently accurate methods to evaluate their stability. This situation is exacerbated on several fronts when screening for appropriate co-formers. First, a separate crystal structure search needs to be performed for each potential co-former. Second, the unit cells of co-crystals are typically significantly larger than those of single-component crystals as quantified by the number of molecules in the unit cell (*Z*) and the number of symmetry independent molecules (*Z*′). This means that there are more degrees of freedom to optimize (*Z*′ > 1), while each energy evaluation is also more expensive (large *Z*). Finally, the stoichiometry of the stable co-crystal is typically unknown, which adds an additional dimension to the search space. As a consequence, computationally efficient and accurate potentials for crystal structure search and co-crystals in particular are highly desirable.

Owing to their outstanding accuracy-to-cost ratio, modern machine-learned (ML) potentials are in principle highly promising in this context. Challenges arise, however, from the importance of long-range contributions due to electrostatics or dispersion. Although recent advances in long-range ML potentials^18−22^ bear good prospect for modeling condensed molecular systems, short-ranged ML potentials are still prevalent and, thus, generally less frequently applied in this context than for gas-phase molecules or ionic solids. As a notable exception, Montes-Campos et al. have nonetheless developed accurate ML potentials for molecular multi-component systems and applied them to the related field of ionic liquids.^23^ In this case, they benefited from the fact that the dynamics of liquids are only weakly influenced by long-range interactions, as is also the case for ion mobilities in solid electrolytes.^24^ The importance of long-range interactions for the relative stabilities of molecular crystal polymorphs is well established, however.^25^

Kapil and Engel overcame this issue by using short-ranged ML potentials for sampling, in combination with additional *ab initio* calculations for stability ranking.^26^ This allowed them to obtain highly accurate thermodynamic stabilities incorporating the combined effects from the electronic structure, quantum nuclear effects, and thermal contributions. In contrast, a Δ-ML^27^ ansatz bypasses the need for subsequent *ab initio* calculations by combining local ML models with appropriate (long-ranged) baselines. This has proven to be highly useful for molecular crystal structure prediction (CSP).^28,29^

**Figure 1:**
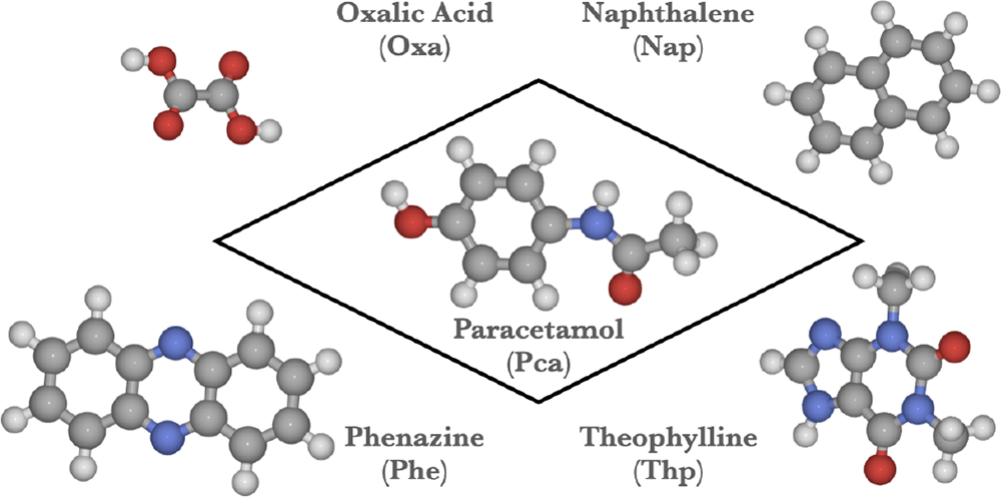
Central active pharmaceutical ingredient paracetamol (Pca) and the co-formers oxalic acid (Oxa), naphthalene (Nap), phenazine (Phe), and theophylline (Thp). Gray spheres: C, blue spheres: N, red spheres: O, white spheres: H.

In a previous study, we presented a framework for the data-efficient generation of Δ-ML models for single-component molecular crystals, which benefits from a separate treatment of inter- and intramolecular interactions.^29^ In this contribution, we present recent advances in extending this approach to co-crystals. Our approach is designed with the co-former screening setting in mind.^30^ Consequently, we will consider a single active pharmaceutical ingredient (paracetamol) combined with four different co-formers, as shown in Figure 1. These systems have been proposed and extensively characterized by Karki et al.^13^ Being one of the most common pharmaceuticals worldwide, paracetamol is a prototypical active pharmaceutical ingredient, while the co-formers oxalic acid (Oxa), naphthalene (Nap), phenazine (Phe), and theophylline (Thp) cover a wide range in terms of polarity, functional groups, and molecular shapes, inducing various types of intermolecular interactions and arrangements in the solid state.

## 2 Methods

### 2.1 General Approach

The approach we previously developed^29^ for single-component crystals has two main features. First, it combines a short-ranged ML potential with a long-ranged physical baseline (Δ-ML). Second, the ML potential is split into an intramolecular and intermolecular correction. The same idea was also used in local approximate models^31^ for lattice energy minimizations of molecular crystals. We found this splitting to be advantageous because these interactions occur on different length scales. Additionally, reference data for the intramolecular correction can be generated cheaply from gas-phase calculations. It is even possible to use a different level of theory for this purpose. Below, we briefly summarize the main points of the method, highlighting the extensions that were developed for co-crystals.

### 2.2 Baseline Method

The dispersion-corrected density functional tight binding (DFTB) method represents an ideal baseline for CSP. First, it is efficient enough to be applied in a setting where several thousands of organic crystal structures need to be optimized.^32^ In addition, the modern third-order variant of DFTB^33^ combined with the 3ob^34^ parameterization provides an accurate description of electrostatics, charge transfer, and polarization. Finally, the missing dispersion contributions can be corrected efficiently, e.g., via the D4 method.^35,36^ The baseline method in this work is thus defined as DFTB3(3ob)+D4 (DFTB+D4 in the following).

### 2.3 Machine Learning Method

The intra- and intermolecular corrections to the baseline will be defined as Gaussian approximation potentials (GAP)^37,61^ using the smooth overlap of atomic position (SOAP)^38^ representation. These GAP models are fitted to both energies and forces. To account for the presence of different molecular building blocks in co-crystals, a separate intramolecular correction is fitted for each. In contrast, a single intermolecular correction is used to describe the interactions among paracetamol and the four co-formers. The energy expression of the combined DFTB+D4 and GAP model (termed Δ-GAP in the following) thus reads
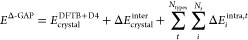
1where for each of the *N*_types_ possible components, the corresponding intramolecular GAP correction is applied to each molecule *i* (in which *N_t_* is the number of molecules of type *t* present in the given unit cell). Note that intra- and intermolecular corrections are applied to energies, forces, and stresses. The models can thus be used for full unit cell relaxations and constant pressure molecular dynamics.

### 2.4 Target Method

The high-level target method to which the correction is fitted will be hybrid DFT (using the PBE0^39^ functional) with a many-body dispersion^25,40^ (MBD) correction. PBE0+MBD provides a sophisticated description of the interactions relevant to organic solids. The importance of MBD contributions and hybrid functionals for the stability assessment of molecular crystals has been highlighted by Hoja and Tkatchenko.^41^ For the X23 database, containing van der Waals (vdW)-bonded, hydrogen-bonded, and mixed molecular crystals, this combination has been shown to yield lattice energies within chemical accuracy (43 meV) when compared to (back-corrected) experimental enthalpies of sublimation.^42^ Moreover, LeBlanc et al. found in their studies on multi-component acid–base crystals that the exact-exchange mixing employed in hybrid DFT is essential to cure significant geometry errors introduced by the delocalization error of semi-local functionals.^43^ Due to the prohibitive computational and memory requirements of PBE0+MBD with large basis sets, we define the target method—called PBE(0)+MBD hereafter—as a composite scheme: The intramolecular part is fully described by PBE0+MBD with a tightly converged basis of numerical atomic orbitals (NAO). The intermolecular part is described by PBE+MBD^44^ with the same basis, plus the difference PBE+MBD to PBE0+MBD in a smaller NAO basis. A similar scheme was used by Hoja et al.,^17^ who found it to yield lattice energies in excellent agreement with converged PBE0+MBD calculations.

### 2.5 Training Data

The structures entering the training set ultimately define the information that is available about the target function. In the context of co-crystal screening studies, the training set should thus include combinations of the molecule of interest with all co-formers. To train the intermolecular model, we selected samples from a pool of ca. 10,000 trial structures created with the PyXtal package.^45^ In this initial pool, a wide range of compositions was considered for each combination to span all possible stoichiometries. These trial candidates were locally relaxed at the DFTB+D4 level of theory. To obtain a diverse set of training structures from this pool, we then employed the farthest point sampling (FPS)^46^ heuristic. Here, the SOAP kernel was used as a similarity measure between atomic environments and structures were sequentially added to the training set by selecting the most dissimilar structures to the current training set at each iteration. Note that there are several possibilities to define global similarity metrics between structures, given a local similarity metric like SOAP.^47^ Herein, we simply used the maximal dissimilarity between any two atomic environments.^48^ From this process, 1000 training structures were obtained, 250 for each crystal/co-former pair (including the corresponding single-component crystals).

We further included 77 structures corresponding to the experimentally known single-component crystals and randomly perturbed structures derived from them. The rationale behind this is that the experimental information about the single-component crystals is usually available in co-crystal studies. This allows us to include some additional information on highly stable interactions, though not for the important paracetamol/co-former contacts. The consequences of this bias in the training set will be discussed in detail below.

In contrast to the intermolecular correction, the training data for the intramolecular model is computationally cheap to generate as it only requires single-point calculations on monomer configurations in the gas phase. To obtain these configurations, monomer geometries were extracted from the training crystals. These were further supplemented, with configurations from gas-phase molecular dynamics simulations and local relaxations, to extensively cover the configurational space of each building block. Further details on the training sets and all training data are provided in the Supporting Information.

## 3 Results and Discussion

**Figure 2:**
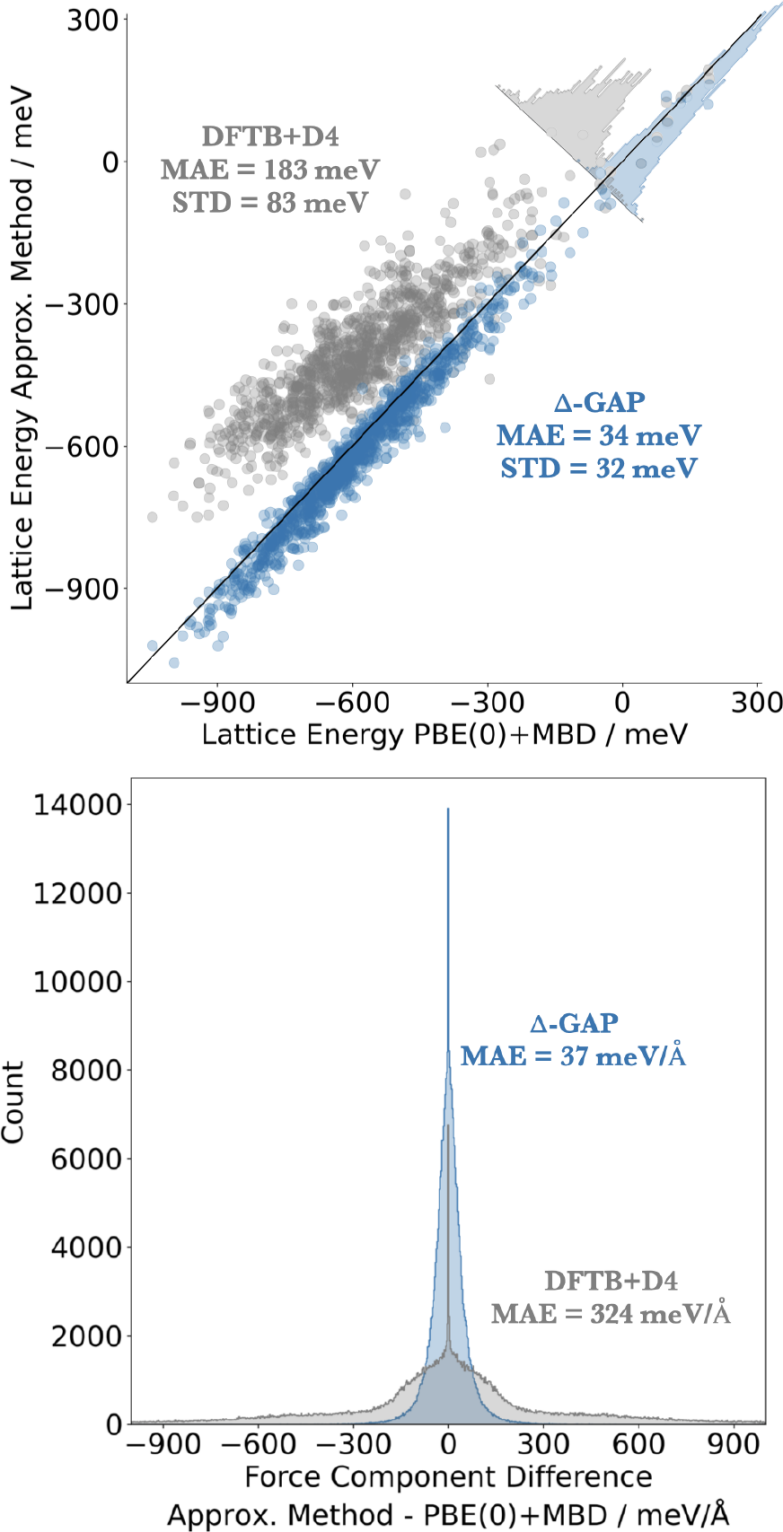
Correlation plot for the DFTB+D4 baseline and Δ-GAP lattice energies per molecule of PcaOxa, PcaNap, PcaPhe, and PcaThp test crystals (both single-component and co-crystals) against the PBE(0)+MBD target level of theory (top) and the corresponding differences in force components (bottom). Note that the slight shift of the Δ-GAP lattice energy distribution toward lower values compared to PBE(0)+MBD is due to the fact that the test set structures are minima on the Δ-GAP potential energy surface, while the training structures are minima on the DFTB+D4 surface (see text). The spike in the distributions of force component differences results from certain force components being zero by symmetry at all levels of theory.

To validate the presented approach, we will first test its performance on a diverse set of crystal structures as one would encounter in a CSP workflow. To this end, a test set of 1000 structures was generated in an analogous procedure to the training set generation. Here, the FPS selection included the training set to maximize the distance between test and training structures (see the Supporting Information for details). All test structures were subsequently relaxed at the Δ-GAP level. Lattice energies and force errors for this test set are summarized in Figure 2. For lattice energy calculation, we used
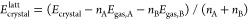
2where the difference between the energy of the crystal, *E*_crystal_, and the energies, *E*_gas_, of its optimized molecular compounds is computed first and then normalized by the total number of compounds in the crystal unit cell. Note that lattice energies of single-component crystals have been calculated in the same way using *n*_B_ = 0.

In Figure 2 (top), Δ-GAP and DFTB+D4 predicted lattice energies are shown in comparison with the PBE(0)+MBD target values. The reference energies cover a broad range of ca. 1 eV per molecule and are mostly negative. This indicates that the random search in general leads to reasonable candidate structures, which are stable with respect to sublimation. The DFTB+D4 lattice energies are reasonably well correlated with this reference but display significant scatter. Furthermore, the lattice energies are systematically underestimated, leading to a mean absolute error (MAE) of 183 meV. Applying intra- and intermolecular corrections to this baseline in the Δ-GAP scheme strongly improves the agreement with the target, resulting in an overall MAE of only 34 meV. This is achieved both by eliminating the systematic underestimation of the lattice energies and by reducing the scatter in the predictions, as indicated by the significantly smaller standard deviation (STD) of the Δ-GAP errors (32 meV vs 83 meV). Indeed, the Δ-GAP energies actually show a slight offset toward more negative values due to the fact that the structures are minima on the Δ-GAP potential energy surface.

An even more substantial improvement is observed for force predictions (see Figure 2, bottom). Here, DFTB+D4 displays a broad error distribution and a correspondingly large MAE of 324 meV/Å. In contrast, the error distribution of predicted Δ-GAP force components is much narrower and the MAE almost an order of magnitude lower. Importantly, while the lattice energy error of DFTB+D4 is fairly systematic, the force error cannot be corrected in a simple way and will lead to substantial deviations in the predicted structures. This is of particular relevance in the context of CSP, where accurate structure relaxations are often by far the most expensive component. Due to their small force errors, Δ-GAP relaxations should provide near PBE(0)+MBD quality structures at a fraction of the computational costs.

While the above results are promising, it should be emphasized that the training and test structures used herein are merely local minima. In particular, they are somewhat less dense and less stable than the known experimental structures for these co-crystals (see the Supporting Information). In future applications, this should be mitigated by using a more advanced CSP search algorithm (ideally together with an accurate ML potential as proposed herein) to generate more realistic structures. From the perspective of this paper, there is also a positive aspect to this discrepancy between training and experimental structures though, as it creates an opportunity to test the extrapolative capabilities of the presented approach. To this end, we test the accuracy of our method on the known experimental structures of each co-crystal.

**Figure 3:**
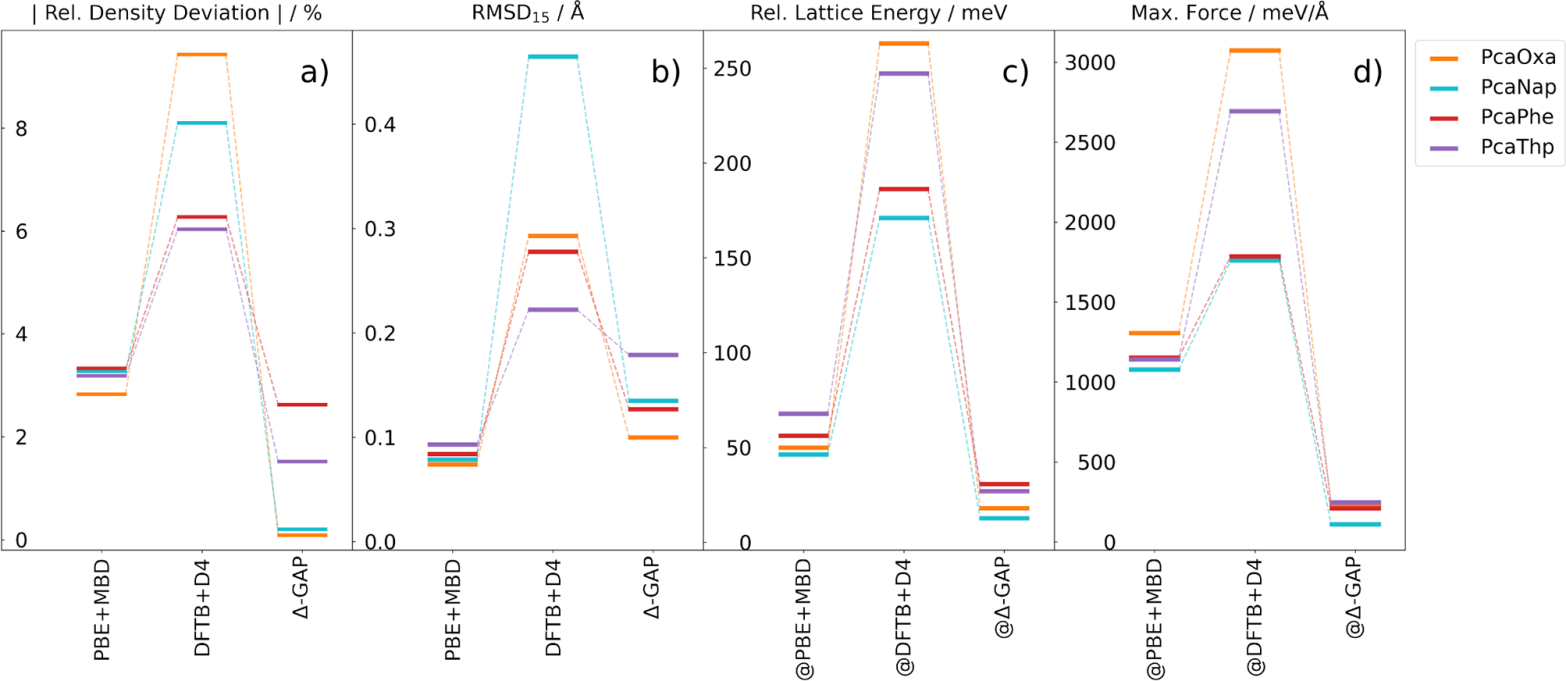
Comparison between PBE+MBD, the DFTB+D4 baseline, and Δ-GAP results on experimental co-crystals for PcaOxa, PcaNap, PcaPhe, and PceThp against the PBE(0)+MBD target level of theory in terms of the absolute values for percentage density deviations (a), the RMSDs between overlaying 15-mers sliced from crystal structures (b), lattice energies per molecule relative to PBE(0)+MBD optimized structures obtained from single-point calculations on structures optimized on the approximate levels of theory specified in the figure (c), and the corresponding maximum remaining PBE(0)+MBD forces (d).

For all experimental co-crystal structures, atomic positions and unit cell parameters were fully relaxed using the DFTB+D4 baseline, Δ-GAP model, and the PBE(0)+MBD target. For comparison, we also performed calculations at the PBE+MBD level, which is often used for relaxations instead of the more expensive hybrid PBE0 functional. These results are summarized in Figure 3.

Relative density deviations with respect to the PBE(0)+MBD geometry are shown in Figure 3a. We find that the DFTB+D4 structures are significantly contracted, in agreement with previous studies where this was attributed to insufficient Pauli-repulsion at longer distances.^32,49^ In contrast, the Δ-GAP structures are in much better agreement, with only slightly higher densities. For comparison, PBE+MBD shows slightly larger but more systematic density deviations of around 3%. In contrast to Δ-GAP and DFTB+D4, this is due to systematically lower densities, which are likely a consequence of differences in the molecular electrostatic potentials predicted by semi-local and hybrid functionals.

**Figure 4:**
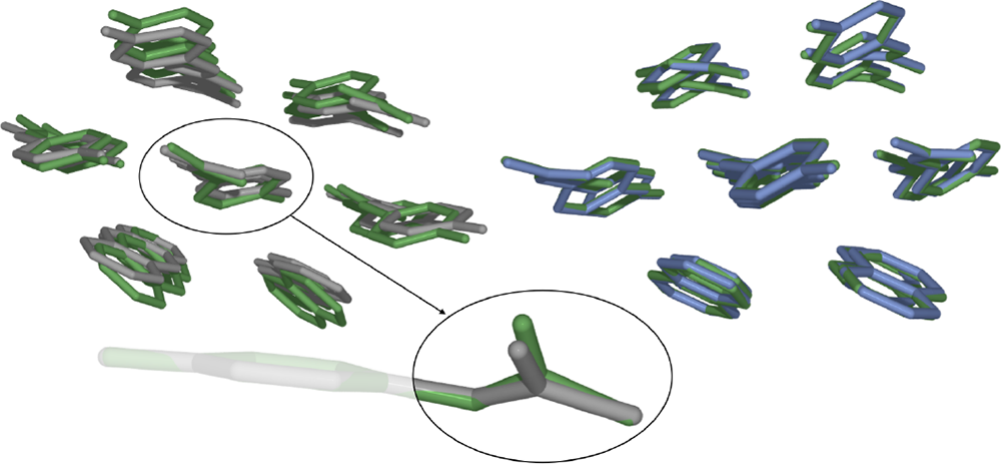
Overlay of the PBE(0)+MBD (green) optimized experimental PcaNap co-crystal with DFTB+D4 (gray) and Δ-GAP (blue). For DFTB+D4, a separate overlay is shown for paracetamol conformers extracted from the crystal environment.

On an atomistic level, crystal structures are typically compared with the RMSD_15_ metric,^50^ as shown in Figure 3b. To this end, the root mean square deviation of the positions of non-hydrogen atoms in 15-molecule clusters extracted from the relaxed crystal structures is calculated. We again use the PBE(0)+MBD structures as the reference. As for the densities, the DFTB+D4 baseline displays the most significant structural discrepancies with the target. These are mostly due to reduced intermolecular distances, such as the spacings in the layered structures PcaOxa, PcaNap, and PcaThp and variations in molecular orientation (see Figure 4 and the Supporting Information for further examples). For PcaNap, additional discrepancy is caused by the intramolecular adjustment of paracetamol to the crystal environment. Here, the DFTB+D4 baseline predicts a weaker out-of-plane rotation of the C=O group, as highlighted in the inset. In all cases, these deviations are mitigated by the ML correction, though the effects are less distinct for PcaThp, which is already reasonably well described by the baseline. Finally, PBE+MBD is slightly more accurate and systematic than Δ-GAP, albeit at a much higher computational cost (by roughly 3 orders of magnitude, see the Supporting Information). Indeed, the structural discrepancies are in this case entirely due to the aforementioned density deviations, whereas the relative positions and orientations of the molecules are in good agreement with the PBE(0)+MBD relaxed structures.

In addition to these geometric comparisons, the relaxed structures were also evaluated from an energetic perspective. This is relevant when structures from the approximate method are used as inputs for single-point calculations or relaxations with higher level methods. Here, small structural deviations—bond distances for instance—can significantly impact predicted energies and energy differences. To evaluate the quality of the structures in this context, single-point PBE(0)+MBD calculations were performed on the geometries predicted by the approximate levels of theory. Figure 3c illustrates the errors in lattice energies obtained from these calculations, while Figure 3d shows the corresponding maximum force. Here, the Δ-GAP values are lowest in all cases, indicating that they are closest to the PBE(0)+MBD minimum from an energetic perspective. The deviations of PBE+MBD are similarly systematic but significantly higher. Finally, the DFTB+D4 results are more scattered and generally poorer with maximum forces of up to 3 eV/Å for the putative minima and lattice energy errors of up to 250 meV.

**Figure 5:**
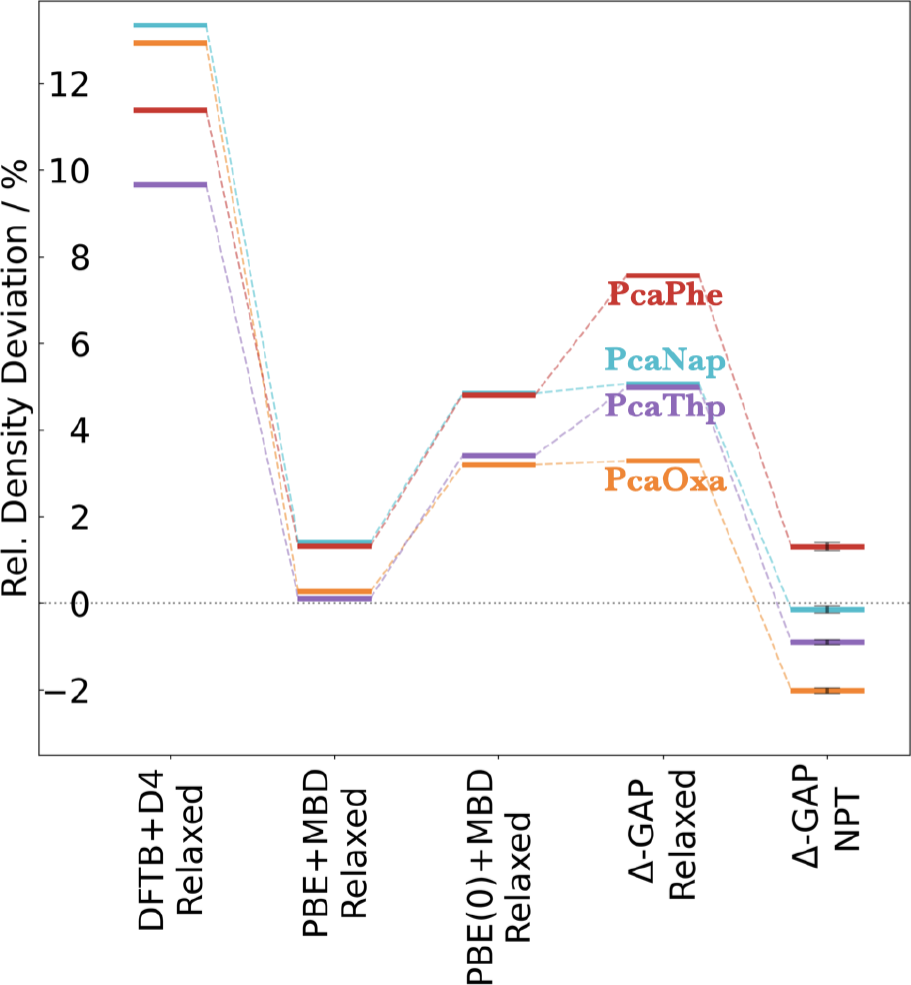
Percentage deviations from experimental measured densities for PcaOxa, PcaNap, PcaPhe, and PceThp co-crystals optimized with the DFTB+D4 baseline, PBE+MBD, the PBE(0)+MBD target level of theory, and Δ-GAP, as well as for densities obtained from Δ-GAP NPT simulations (298 K and 1 bar). For NPT, results corresponding to standard errors of the deviations are illustrated.

Overall, the Δ-GAP model is thus a robust and significant improvement on DFTB+D4, even when applied outside the range of the training set. Perhaps surprisingly, it is even an improvement over the much more expensive PBE+MBD method in many respects, when comparing with the PBE(0)+MBD target. Of course, the ultimate test is comparison with experimental structures, however. Here, we somewhat unexpectedly found that the PBE+MBD densities are actually closer to the experimental values than the ones predicted by PBE(0)+MBD (and consequently also by Δ-GAP, see Figure 5).

These apparent deviations can be resolved by considering thermal effects, however. Computationally relaxed crystal structures correspond to the 0 K limit, whereas crystallographic experiments are usually performed at finite temperature and pressure. The over-contraction of PBE(0)+MBD will thus be counteracted by thermal expansion. An advantage of computationally efficient approaches like Δ-GAP is that they allow for including such effects in a straightforward manner by performing molecular dynamics in the NPT ensemble (at 298 K and ambient pressure). As shown in Figure 5, the average densities across these trajectories are indeed in very good agreement with the experiment. This also indicates that the PBE+MBD (0 K) densities are in fact fortuitously close to the experiment as the inclusion of thermal expansion effects would likely also cause them to decrease by ca. 5%.

Importantly, such finite temperature simulations would be computationally prohibitive on the hybrid DFT level. Being an efficient surrogate for PBE(0)+MBD, Δ-GAP thus allows performing simulations that would otherwise be impossible. These results also further underscore the robustness of our ML approach, given that the experimental structures are outside the scope of the training set and no crystal MD data was used for training at all. This is thanks to the strong physical prior that the DFTB+D4 baseline provides and the smoothness of the GAP correction. Additional improvements could be obtained by combining the current approach with more advanced structure search algorithms^51−53^ and by iteratively refining the GAP correction in an active learning workflow.

## 4 Conclusions

We have presented an approach for Δ-ML potentials applicable to both pure crystals and co-crystals of variable composition. This Δ-GAP approach enables efficient global crystal structure searches with near hybrid DFT accuracy, at a much reduced cost. Building on a previous approach for single-component crystals, we fit separate intramolecular corrections for each component and a single intermolecular correction for all active molecule/co-former pairs. Our approach strongly reduces energy and force errors with respect to the baseline model.

Notably, the training structures used herein were generated with a simple random search procedure and consequently display markedly lower densities and stabilities than the known experimental co-crystals. Nevertheless, the Δ-GAP potentials are able to predict the structures of experimental polymorphs with high accuracy, outperforming PBE+MBD at a much lower computational cost. This shows that this approach is highly robust in an extrapolative regime. In future work, we aim to combine these potentials with more advanced CSP search algorithms.^51−53^

Finally, it should be noted that many-body dispersion can be rather long-ranged in some cases,^54^ while our baseline method relies on the D4 correction, which lacks these effects. Since the intermolecular ML contributions are by construction short-ranged due to the use of a local representation, long-range many-body dispersion effects are thus currently neglected in our approach. This could be mitigated by including a physical many-body dispersion model in the baseline. An efficient ML-based MBD implementation that makes this computationally feasible has recently been reported.^55,56^

## 5 Computational Details

DFT calculations were performed with the all-electron code FHI-aims,^57^ using the PBE^44^ and PBE0^39^ functionals. A post-SCF dispersion correction was applied using the MBD^25,40^ method. Two accuracy levels with a large or small basis set have been used (compare Section 2). Large basis set calculations correspond to tier2 settings and tight integration grids, while small basis set calculations correspond to tier1 settings and light integration grids. DFTB3^33^ calculations were performed using DFTB+^58^ together with the 3ob^34^ parametrization and the D4^35,36^ dispersion correction without non-additive effects. For periodic calculations, the number of **k** points (*n*) in each direction is chosen as the smallest integer satisfying the relation *n*·*a* ≥ *x*, where *a* is the unit cell length along that direction and *x* = 30. GAP potentials were trained and evaluated using the QUIP^59^ package. Candidate crystal structures were created with the PyXtal^45^ package.

## Supporting Information Available

Method and additional details on co-crystal stabilities, density dependence of the lattice energies for experimental co-crystals, structural overlay of optimized experimental co-crystals, density and lattice energy comparison between training and experimental co-crystals, molecular dynamics simulations, and comparison of computational costs (PDF)
