# Supplementary material for: A Hybrid Machine Learning Approach for Structure Stability Prediction in Molecular Co-crystal Screenings
Source: J Chem Theory Comput. 2022 Jun 16;18(7):4586–93. doi: 10.1021/acs.jctc.2c00343 (PMC9281391; doi:10.1021/acs.jctc.2c00343)
Supplement: Supplementary file 1 — ct2c00343_si_001.pdf [file ct2c00343_si_001.pdf]

# Supplementary Information on A Hybrid Machine Learning Approach for Structure Stability Prediction in Molecular Co-Crystal Screenings

Simon Wengert,[1,2] Gábor Csányi,[3] Karsten Reuter,[1] and Johannes T. Margraf[1,a)]

[1)] *Fritz-Haber-Institut der Max-Planck-Gesellschaft, Faradayweg 4-6, 14195 Berlin, Germany*

[2)] *Chair of Theoretical Chemistry, Technische Universität München, 85747 Garching, Germany*

[3)] *Engineering Laboratory, University of Cambridge, Cambridge CB2 1PZ, United Kingdom*

[a)] Electronic mail: johannes.margraf@ch.tum.de

# I. METHOD

Complementing the energy expression for the combined DFTB+D4 and GAP model presented in the main text, Eqs. 2 and 3 describe the analog expressions for forces and stresses. In order to evalutate the stress correction with the intramolecular model, each of the molecules is successively placed in the unit cell as depicted in Fig. S1. Evaluation of the model on each of these structures delivers their intramolecular stress correction contribution. In case periodic replicas of a molecule fall into the local environment of the central one—defined by the cutoff value of the SOAP descriptor—this would lead to wrong values, however. To avoid this, the unit cell is expanded until all periodic replica are outside the cutoff region. The obtained stress correction for these structures is then multiplied with a prefactor $\mathrm{f_e}$ which back-corrects this expansion.

$$E^{\Delta-\mathrm{GAP}} = E^{\mathrm{DFTB+D4}}_{\mathrm{crystal}} + \Delta E^{\mathrm{inter}}_{\mathrm{crystal}} + \sum_{t}^{N_{type}} \sum_{i}^{N_t} \Delta E_i^{\mathrm{intra},t} \tag{1}$$

$$\mathbf{F}^{\Delta-\mathrm{GAP}} = \mathbf{F}^{\mathrm{DFTB+D4}}_{\mathrm{crystal}} + \Delta \mathbf{F}^{\mathrm{inter}}_{\mathrm{crystal}} + \sum_{t}^{N_{type}} \sum_{i}^{N_t} \Delta \mathbf{F}_i^{\mathrm{intra},t} \tag{2}$$

$$\mathbf{S}^{\Delta-\mathrm{GAP}} = \mathbf{S}^{\mathrm{DFTB+D4}}_{\mathrm{crystal}} + \Delta \mathbf{S}^{\mathrm{inter}}_{\mathrm{crystal}} + \sum_{t}^{N_{type}} \sum_{i}^{N_t} \mathrm{f_e} \cdot \Delta \mathbf{S}_i^{\mathrm{intra},t} \tag{3}$$

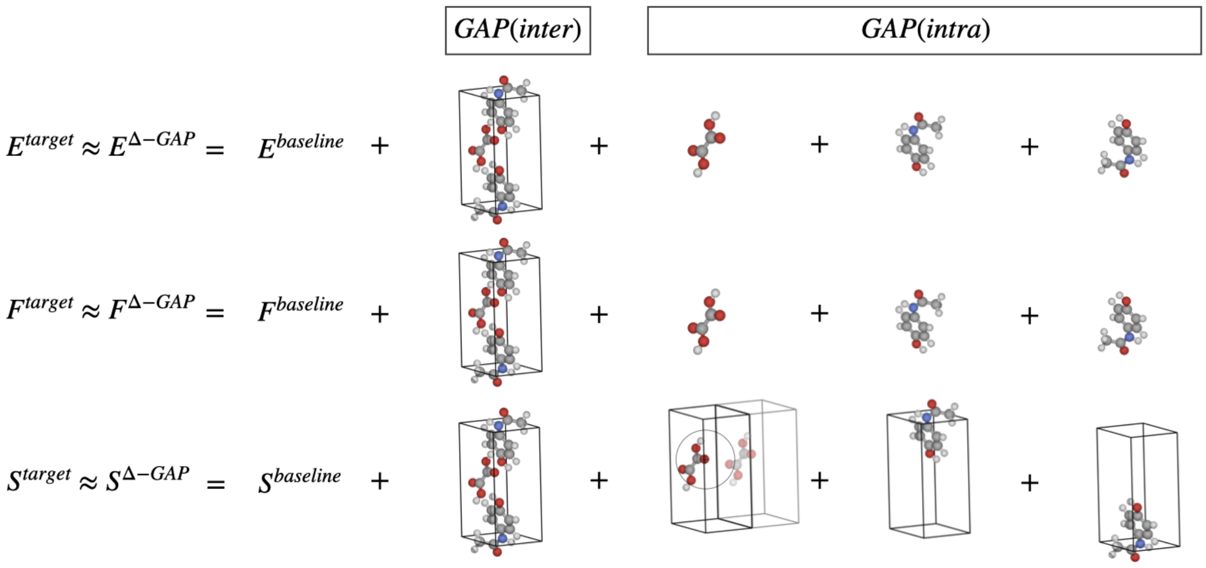


FIG. S1: Visual representations of energy, force and stress expressions for approximating the PBE(0)+MBD target level of theory with the combined DFTB+D4 baseline and GAP model (denoted by Δ-GAP).

## II. RESULTS AND DISCUSSIONS

### A. Co-Crystal Stabilities

In the main document we discuss the agreement in terms of lattice energies obtained on the test set between the DFTB+D4 baseline, the Δ-GAP model and the PBE(0)+MBD target. While the corresponding correlation plot illustrates the predictive power in a compact way by capturing the various stochiometries of all four co-crystals systems, it does not allow for a direct comparison of crystal stabilites as conducted in CSP studies. Here, the energetic ordering of crystal structures is arguably of greater importance than the numerical value of the lattice energy—which might be subject to systematic deviations. For this reason, we provide a comparison of ranking orders in Fig. S2 focusing on the stoichiometries identical to the experimentally known co-crystals. It can be seen that even in absence of systematic deviations applying the ML correction significantly improves the stability assessment. The Spearman's rank correlation coefficient ($r_s$) assesses monotonic relationships between data sets and is used to compare the ranking orders of PBE(0)+MBD and the approximate methods. It provides correlation in a range between -1 (opposite ordering) and +1 (identical

ordering). With Δ-GAP values consistently larger compared to DFTB+D4 and in a range close to 1 our findings underscore the capability of Δ-GAP to improve the detection of promising structure candidates during CSP studies.

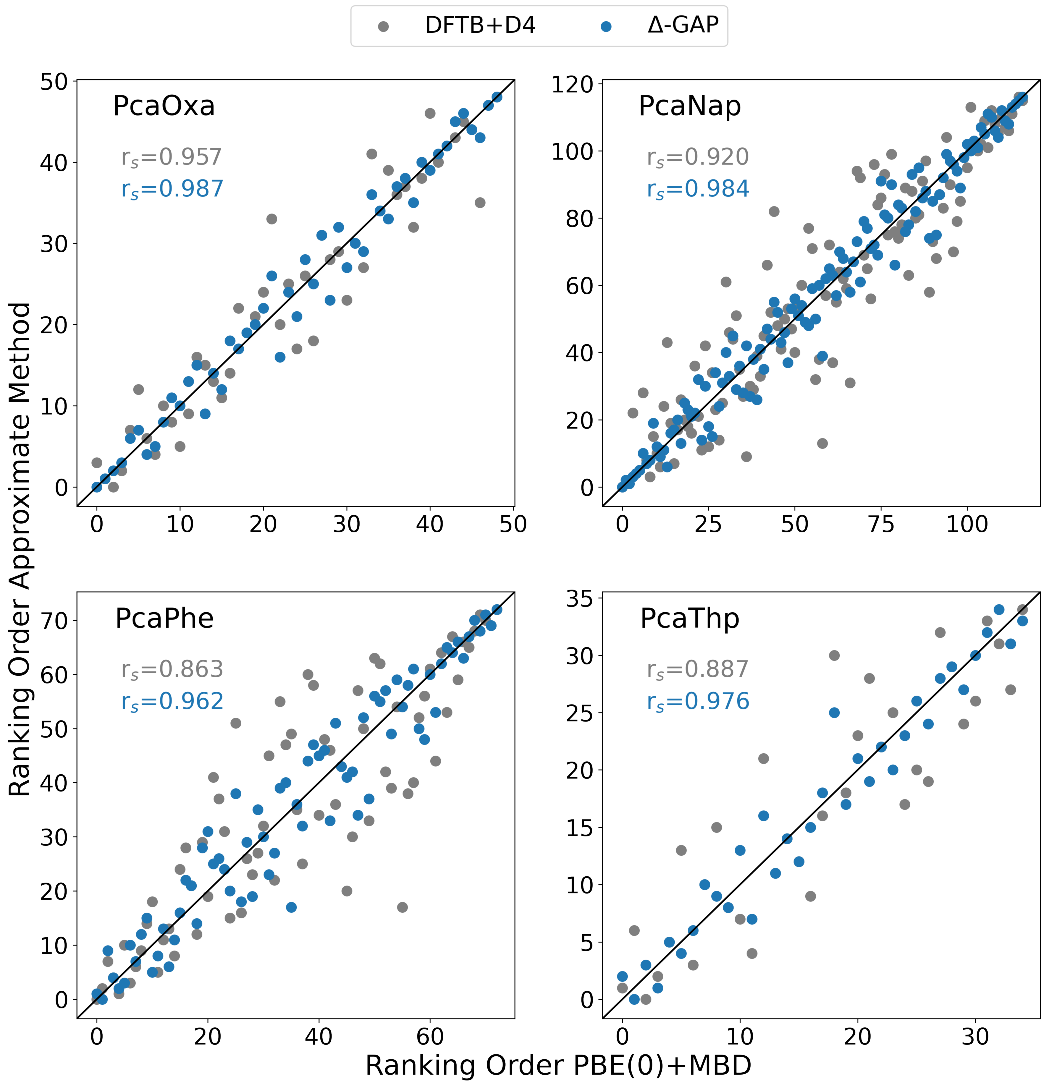


FIG. S2: Correlation between ranking orders obtained from PBE(0)+MBD lattice energies and the two approximate methods DFTB+D4 and Δ-GAP for test set structures with identical stoichiometry as their experimental counterparts.

## B. Density-dependence of Lattice Energies for Experimental Co-Crystals

To test the accuracy of our method on the known experimental structures of each co-crystal we start by conducting full unit cell relaxations on the different levels of theory. As full unit cell relaxations are not possible at the PBE(0)+MBD level, we start from the PBE+MBD cell in this case. Subsequently, atomic positions are relaxed for scaled variants of this cell, in order to obtain a good approximation of the relaxed PBE(0)+MBD density. Analogous scans were also performed with DFTB+D4 and Δ-GAP, around their respective minima. The corresponding energy vs. density curves are shown in Fig. S3.

Here, the difference between the DFTB+D4 baseline and the other two methods is striking. In particular, the lattice energies and minimum densities are significantly too high. Δ-GAP and PBE(0)+MBD, on the other hand, mostly agree well in their descriptions of these high-stability regions. Here, the largest deviation is the Δ-GAP curve for the PcaThp minimum, which is somewhat deeper than the PBE(0)+MBD target. The location of the minimum appears at comparable density though, which holds true across all four co-crystal systems. These finding show that besides the locations of the minimum itself Δ-GAP provides an adequate description also of the region around them when compared to the high-level PBE(0)+MBD results. Since these regions correspond to structures outside the training samples it further underscore the robustness of our method.

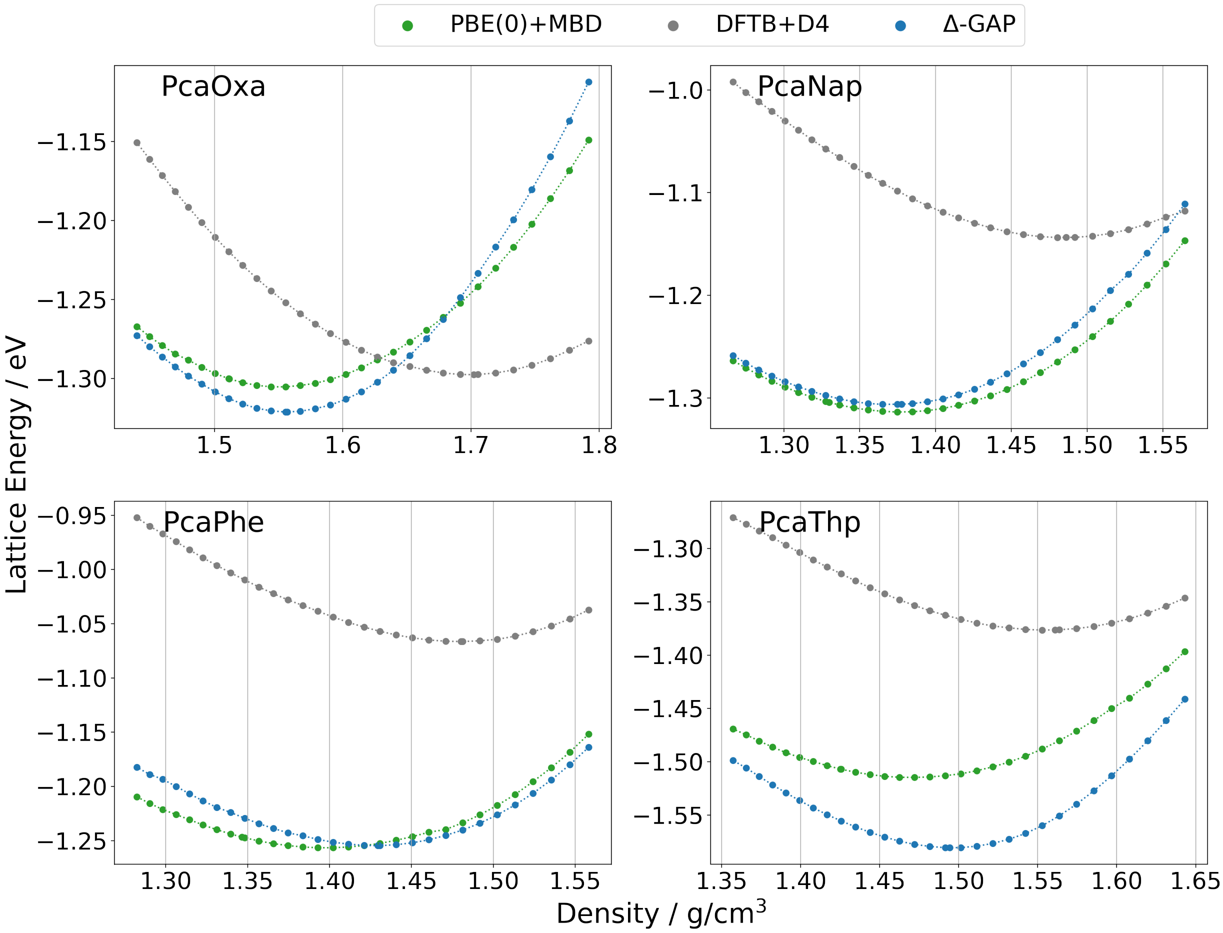


FIG. S3: Density scan for lattice energies per molecule around experimental co-crystal structures of PcaOxa, PcaNap, PcaPhe and PcaThp optimized with the DFTB+D4 baseline, Δ-GAP and the PBE(0)+MBD target level of theory.

## C. Structural Overlay of Optimized Experimental Co-Crystals

For the experimental co-crystals PcaOxa, PcaPhe and PcaThp relaxed with the DFTB+D4 baseline, Δ-GAP and the PBE(0)+MBD target level of theory Figs. S4, S5 and S6 illustrate corresponding overlays of non-hydrogen atoms in 15-molecule clusters (complementary to the PcaNap overlay shown in the main document). Reduced intermolecular distances as obtained with the DFTB+D4 baseline can be seen, in particular from the spacing for the layered structures PcaOxa and PcaThp. For each of the three co-crystals structural

deviations to the PBE(0)+MBD target are mitigated by applying the ML correction.

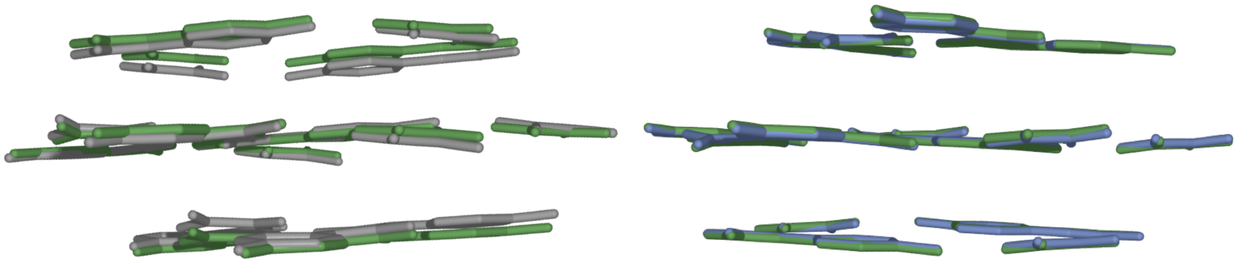

FIG. S4: Overlay of PBE(0)+MBD (green) optimized experimental PcaOxa co-crystal with DFTB+D4 (gray) and Δ-GAP (blue)

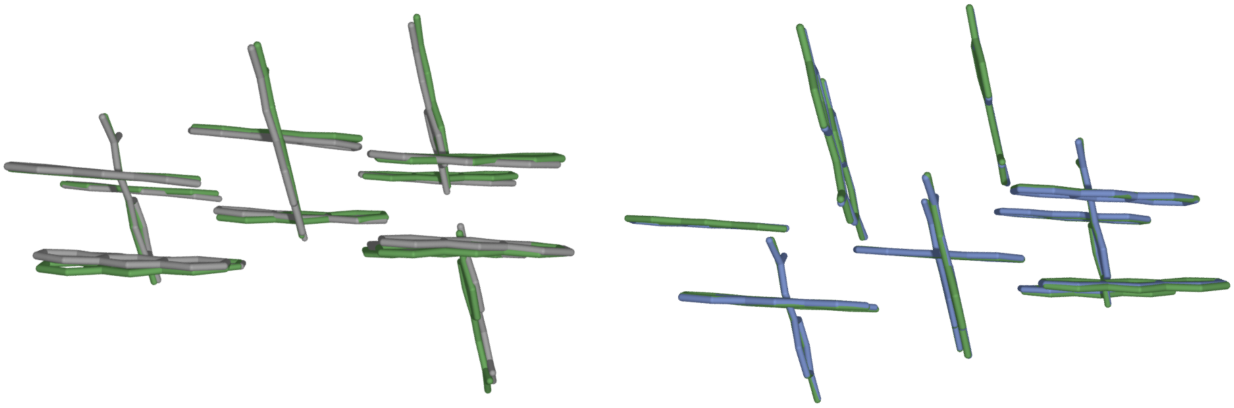

FIG. S5: Overlay of PBE(0)+MBD (green) optimized experimental PcaPhe co-crystal with DFTB+D4 (gray) and Δ-GAP (blue)

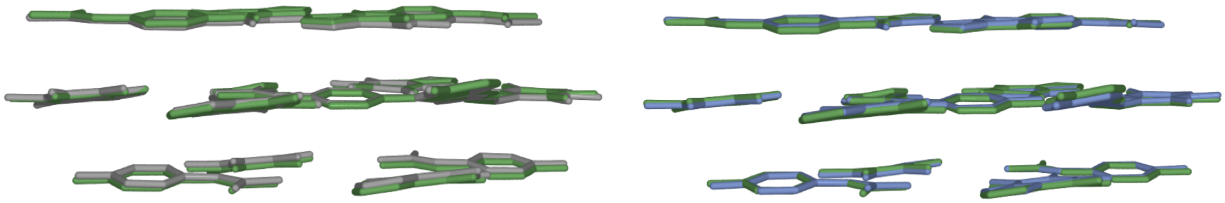

FIG. S6: Overlay of PBE(0)+MBD (green) optimized experimental PcaThp co-crystal with DFTB+D4 (gray) and Δ-GAP (blue)

### D. Density and Lattice Energy Comparison Between Training and Experimental Co-Crystals

As mentioned in the main manuscript, the employed random structure generation yields less dense and less stable co-crystals compared to the ones experimentally known. Here, we want to provide some more detailed insight into this by directly comparing the experimental co-crystals with structures of identical stoichiometry used to train the $\Delta$-GAP model. The comparison in terms of densities is illustrated in Fig. S7, while Figs. S8 and S9 are concerned with the lattice energies obtained with DFTB+D4 and PBE(0)+MBD. Note that for both methods lattice energies of the experimental co-crystals correspond to structures optimized on the respective level of theory. Training structures, on the other hand, are always optimized with the DFTB+D4 baseline. Although lattice energies of training structures are therefore in better agreement with experimental co-crystals in Fig. S8, a gap still persists that is to be mitigated by a more advanced structure search algorithm in the future.

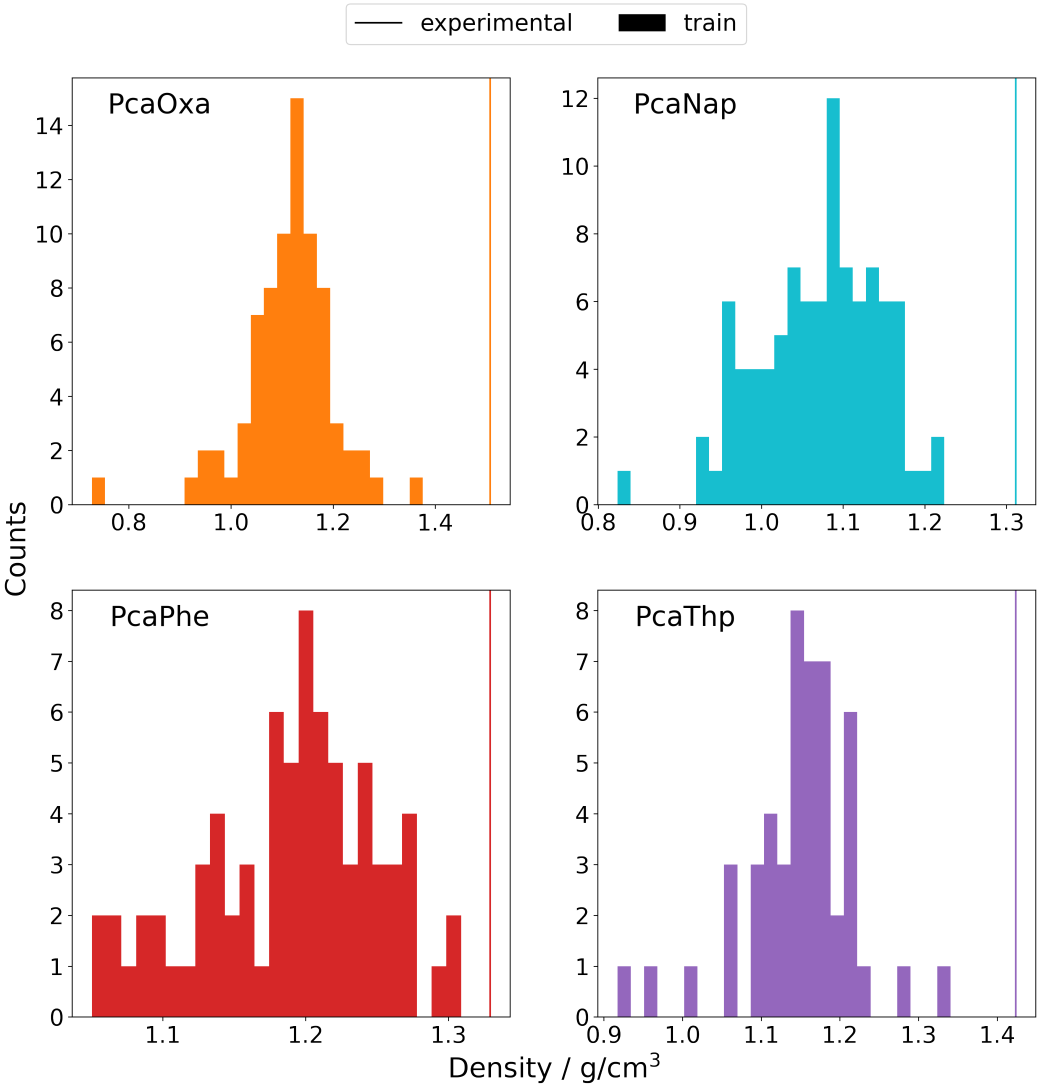


FIG. S7: Densities of the experimental co-crystals PcaOxa, PcaNap, PcaPhe and PcaThp together with density distributions of training structures with identical stoichiometry as their experimental counterparts.

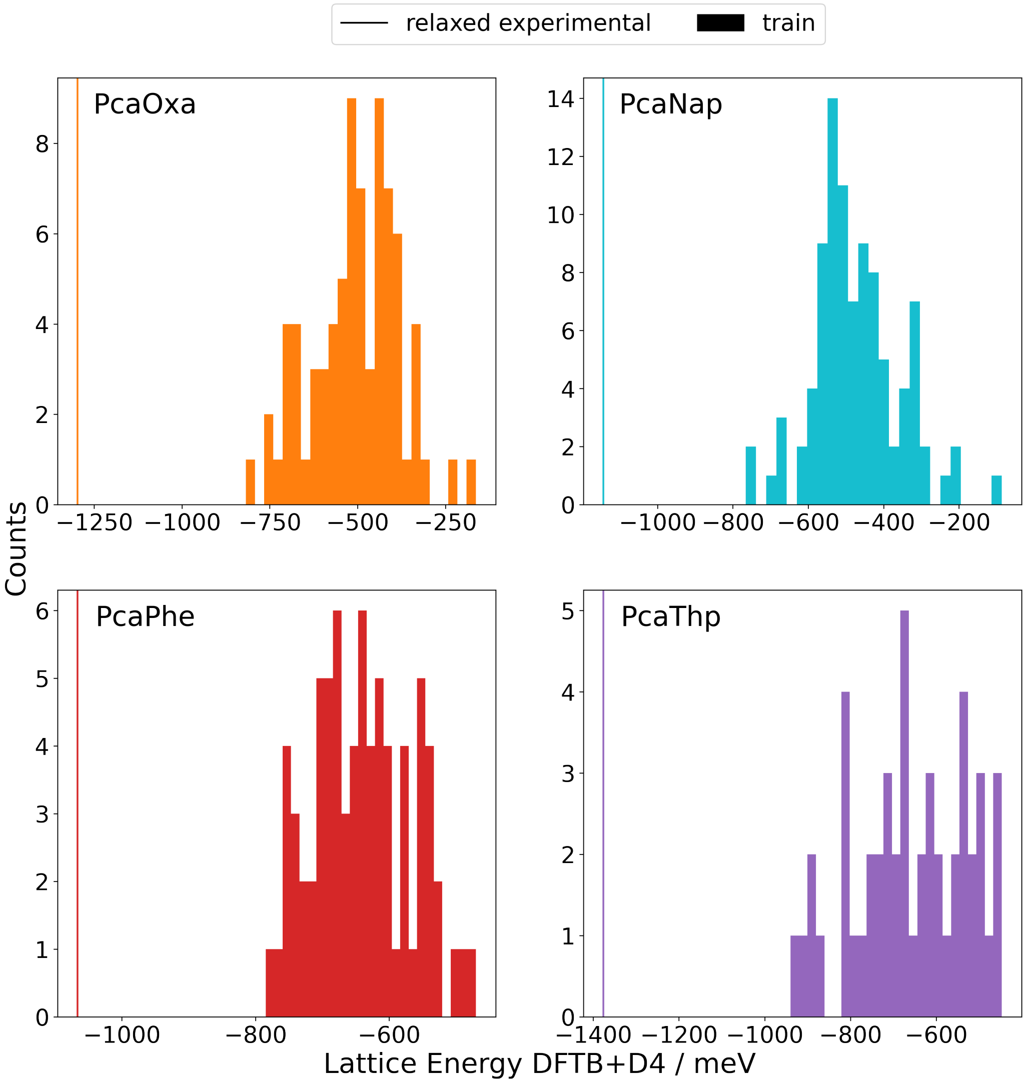


FIG. S8: DFTB+D4 lattice energies per molecule of the relaxed experimental co-crystals PcaOxa, PcaNap, PcaPhe and PcaThp together with lattice energy distributions of training structures with identical stoichiometry as their experimental counterparts.

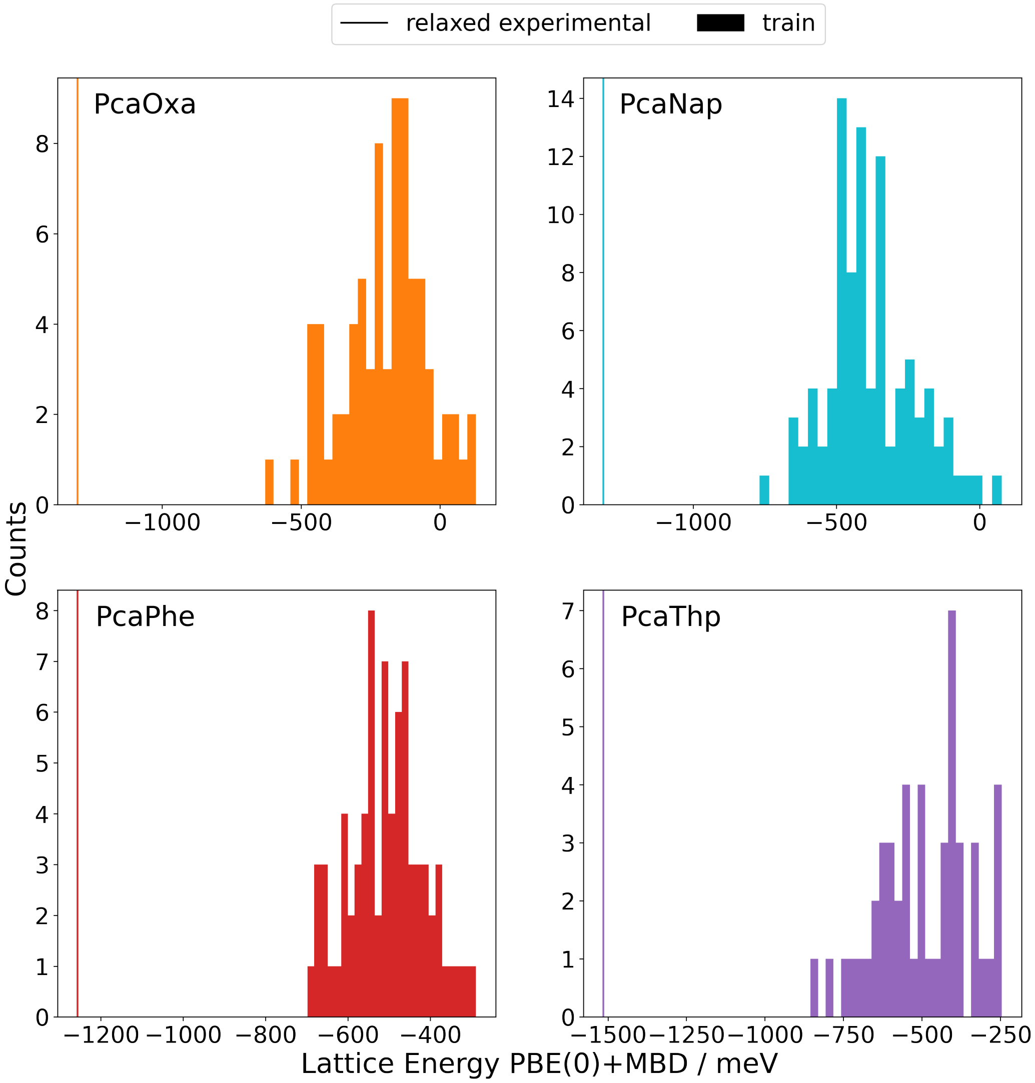


FIG. S9: PBE(0)+MBD lattice energies per molecule of the relaxed experimental co-crystals PcaOxa, PcaNap, PcaPhe and PcaThp together with lattice energy distributions of training structures with identical stoichiometry as their experimental counterparts.

## E. Molecular Dynamics Simulations

For molecular dynamics simulations at constant pressure (1 bar) and temperature (298 K), $2 \times 2 \times 2$ supercells of the $\Delta$-GAP optimized experimental co-crystals PcaOxa, PcaNap, PcaPhe and PcaThp where used. After an equilibration phase, we used data from 122.9 ps (PcaOxa), 90.4 ps (PcaNap), 98.5 ps (PcaPhe) and 33.4 ps (PcaThp) trajectories for calculating the mean densities, as well as the related standard errors. Note that the shorter simulation time for PcaThp is due to the large number of atoms in its supercell (1312 atoms), which makes each step more expensive compared to the other three systems (PcaOxa: 896 atoms, PcaNap: 928 atoms, PcaPhe: 1024 atoms). Nonetheless, the mean densities are well converged in all cases, as indicated by the corresponding standard errors in Table S1. Standard errors were calculated with the number of statistically independent snapshots, as estimated from the density autocorrelation functions.

| System | Mean Density / $g/cm^3$ | Standard Error / $g/cm^3$ |
|---|---|---|
| PcaOxa | 1.48 | 0.0009 |
| PcaNap | 1.31 | 0.0010 |
| PcaPhe | 1.35 | 0.0012 |
| PcaThp | 1.41 | 0.0008 |

TABLE S1: Mean densities obtained from NPT simulations of experimental co-crystals together with corresponding standard errors.

## F. Comparison of Computational Costs

To provide an estimate for the relation of computational costs for the individual methods CPU-times obtained for the experimental PcaThp crystal have been analysed. For the relations between DFTB+D4 : $\Delta$-GAP : PBE+MBD : PBE(0)+MBD we find an increase in computational costs according to 1:2:3200:15000. The corresponding calculations were performed on Intel Xeon IceLake Platinum 8360Y processors with a base frequency of 2.4 GHz.
